# Fatal Differentiation Syndrome Complicating Acute Promyelocytic Leukemia Treatment: A Case Report

**DOI:** 10.7759/cureus.54491

**Published:** 2024-02-19

**Authors:** Umair Khizer, Bhavana Annam, Akasha Akhtar, Jasninder S Dhaliwal, Chieh Yang

**Affiliations:** 1 Internal Medicine, University of California Riverside School of Medicine, Riverside, USA; 2 Internal Medicine, Saint Luke's Health System, Kansas City, USA

**Keywords:** all-trans retinoic acid, steroids for differentiation syndrome, arsenic trioxide, acute promyelocytic leukemia, differentiation syndrome

## Abstract

We report the case of a 42-year-old female diagnosed with acute promyelocytic leukemia (APL), who developed differentiation syndrome (DS) on day 14 during induction therapy with all-trans retinoic acid (ATRA) and arsenic trioxide (ATO) with sudden-onset dyspnea, abdominal pain, tachycardia, and fever. Her laboratory findings were remarkable for acute kidney injury (AKI), worsening leukocytosis, thrombocytopenia, and lactic acidosis. She was also found to have flash pulmonary edema and a pericardial effusion. Despite immediate dexamethasone and methylprednisolone administration along with cessation of induction therapy, she continued to worsen and suffered a non-shockable cardiac arrest. Return of spontaneous circulation (ROSC) was achieved, but she was in profound shock requiring multiple vasopressors. The patient suffered repeat cardiac arrest later that day and passed away within 24 hours. DS is a potentially life-threatening complication in APL treatment, occurring in about 25% of APL patients and posing significant treatment challenges.

## Introduction

Acute promyelocytic leukemia (APL) is a distinct variant of acute myeloid leukemia (AML) that is characterized by balanced translocation t(15;17)(q24.1;q21.1) involving the retinoic acid receptor alpha (RARA) gene on chromosome 17 and the promyelocytic leukemia (PML) gene on chromosome 15 resulting in a fusion gene known as PML-RARA [1]. This arrests the differentiation of leukemic cells at the promyelocytic stage. Treatment for APL involves all-trans retinoic acid (ATRA) and arsenic trioxide (ATO) which promotes the differentiation of promyelocytes into mature myeloid cells [2]. The incidence of differentiation syndrome (DS) in APL patients being treated with ATRA and ATO can range from 2% to 48%. The mechanism of DS is not fully understood but involves a large number of maturing myeloid cells that produce inflammatory cytokines and infiltrate tissues including the lymph nodes, spleen, liver, pericardium, and lungs [3]. This case report presents a rare case of fatal DS that led to rapid decompensation and death despite dexamethasone and methylprednisolone administration along with cessation of ATRA and ATO therapy.

## Case presentation

A 42-year-old morbidly obese female with a body mass index of 46 presented to the emergency department (ED) with complaints of heavy vaginal bleeding for three days. The patient stated she had been having heavy menstrual bleeding for three years and had been on medroxyprogesterone on and off, but this episode was severe leading to dizziness. She described dizziness as if she was going to pass out. She also complained of generalized fatigue and bruising on the legs for a week. Her vitals showed a temperature of 36.8°C (oral), heart rate of 82 beats per minute (bpm), blood pressure of 147/73 mmHg, and oxygen saturation of 97% on room air. Physical examination showed conjunctival pallor, cervical lymphadenopathy, and scattered bruising on bilateral arms and legs. Her complete blood count showed pancytopenia which is summarized in Table 1. Serum electrolytes, kidney function, and liver function were normal. Serum troponin was 0.759 ug/mL which later decreased to 0.304 ug/mL and was deemed to be due to demand ischemia of the heart from anemia. The pregnancy test was negative. Chest X-ray showed no acute abnormality. An electrocardiogram (ECG) showed sinus tachycardia and normal QT interval. Transthoracic echocardiogram (TTE) showed mild eccentric left ventricular (LV) hypertrophy with left ventricular ejection fraction (LVEF) of 60-65% and pulmonary artery systolic pressure (PASP) of 54 mmHg. 

**Table 1:** Complete blood count with differentials

Laboratory value	Result	Normal range
White blood cells	1.6	4-11x10^3^/µL
Red blood cells	2.01	4.6-6.1x10^6^/µL
Hemoglobin	6.7	12-16 g/dL
Hematocrit	19.5	36-46%
Mean corpuscular volume	97	80-100 fL
Mean corpuscular hemoglobin	33.6	27-32 pg
Mean corpuscular hemoglobin concentration	34.6	32-36 g/dL
Red blood cell distribution width	22.2	11.5-15.5%
Platelets	33	150-450x10^3^/µL
Mean platelet volume	9.4	7-10 fL
Segmented neutrophils	20	45-70%
Lymphocytes	54	20-40%
Monocytes	2	3-8%
Myelocytes	4	0-5%
Neutrophil abnormalities	0.3	1.5-8x10^3^/µL
Nucleated red blood cells	12	≤5 per 100 white blood cell
Haptoglobin	55	50-220 mg/dL
Retic count	5.98	0.5-2.5%
Lactate dehydrogenase	395	120-221 units/L

**Figure 1:**
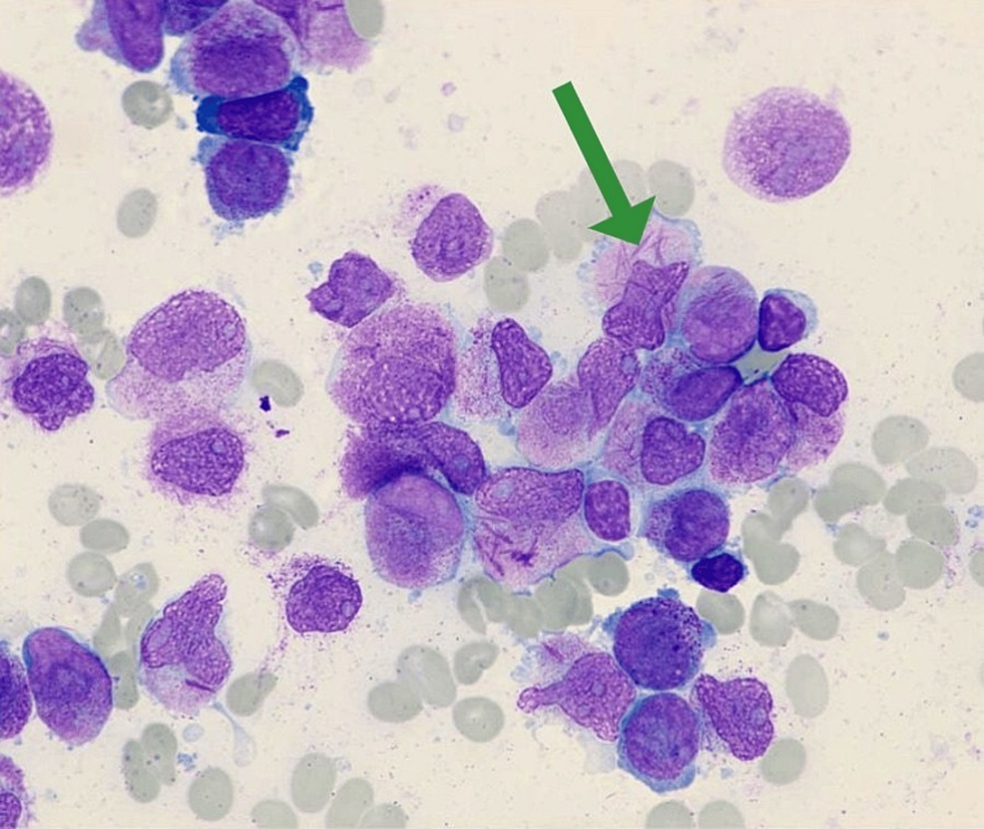
A sample of acute promyelocytic leukemia peripheral blood smear showing promyelocyte (green arrow) with Auer rods

**Figure 2:**
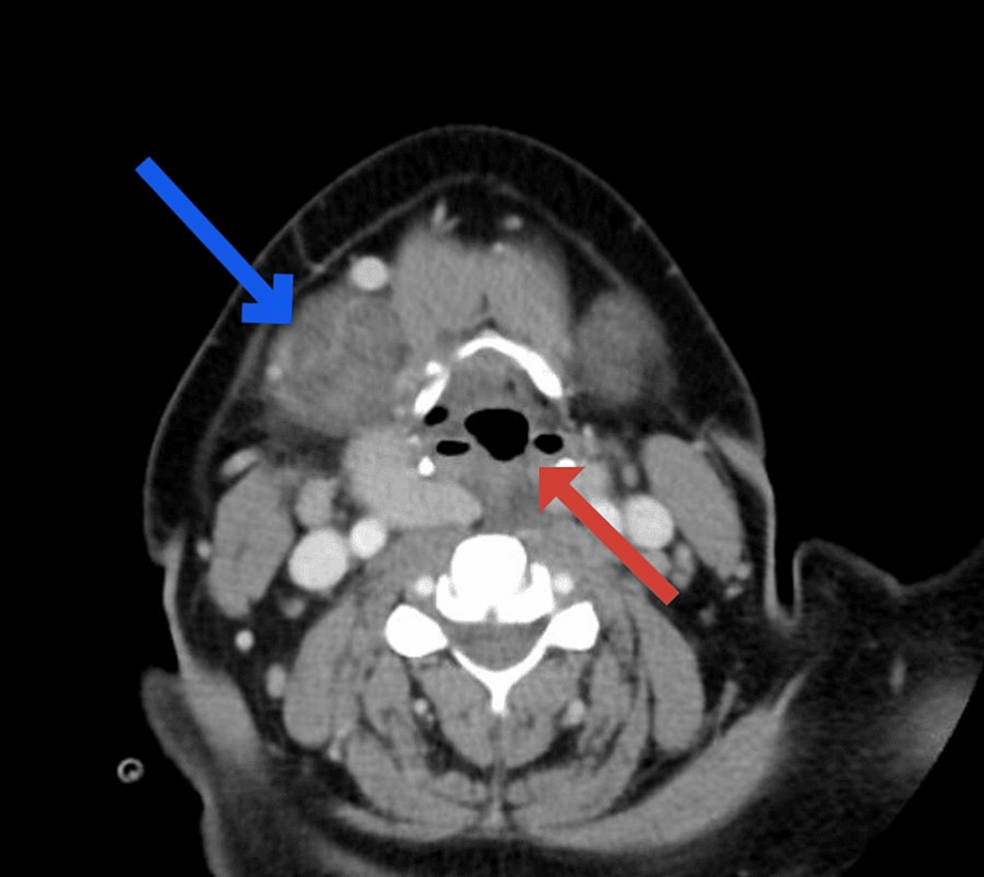
Enlarged submandibular gland (blue arrow) and palatine tonsil (red arrow)

The patient's coagulation profile was consistent with disseminated intravascular coagulation (DIC) with elevated prothrombin time (PT), elevated partial thromboplastin time (PTT), thrombocytopenia, and decreased fibrinogen which is summarized in Table 2. Two packs of red blood cells (pRBCs) and one unit of platelets were transfused in the ED. Obstetrics-Gynecology was consulted, and medroxyprogesterone was started for her vaginal bleeding. Pelvic ultrasound showed no abnormalities. Peripheral blood smear showed acute leukemia with circulating blasts/immature cells, promyelocytes (Figure 1), marked normocytic anemia, and marked thrombocytopenia. Flow cytometry of peripheral blood was consistent with APL. The patient had a computed tomography (CT) angiogram of the abdomen and pelvis that showed no acute pathology explaining active pelvic bleeding. No pelvic lymphadenopathy was seen. CT scan of the chest, abdomen, and pelvis was normal. The patient started complaining of dysphagia, and a CT scan of the soft tissues of the neck was obtained that showed enlargement of the palatine tonsils and submandibular gland (Figure 2). The patient was started on piperacillin-tazobactam for tonsillitis with immunocompromised status.

**Table 2:** Coagulation profile

Test	Value	Normal range
Prothrombin time	19.2	11.5-14.5 sec
Partial thromboplastin time	38.2	23-35 sec
International normalized ratio	1.5	0.9-1.1
Fibrinogen	126	200-450 mg/dL
D-dimer	>20	<0.50 ng/mL
Platelet count	54	150-450x10^3^/uL

**Figure 3:**
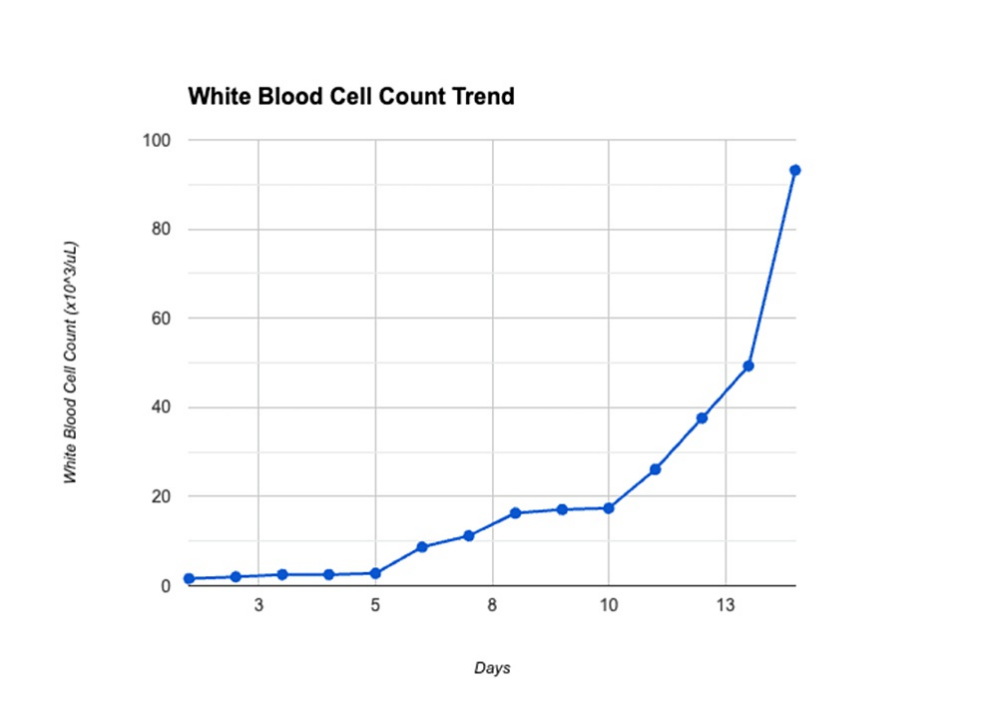
White blood cell count trend

Hematology-Oncology was consulted, and the patient was started on chemotherapy for APL with ATRA 56 mg orally twice a day (standard dose mg/m^2^/day) and ATO 20 mg intravenous (IV) daily (standard dose 0.15 mg/kg/day) along with 100 cc/hr of normal saline. A bone marrow biopsy eventually showed APL with promyelocytes and normocytic pancytopenia. Fluorescence in situ hybridization (FISH) test on blood sample showed PML-RARA fusion product. On day 4 of chemotherapy, her platelets worsened to 20,000/µL, and her international normalized ratio (INR) increased to 1.6. She started complaining of gingival bleeding while brushing her teeth, and two additional units of platelets were given along with four units of fresh frozen plasma (FFP). Her white blood cell (WBC) count continued to increase daily. The sequential increase is shown in Figure 3. Infectious Disease (ID) was consulted, and her leukocytosis was deemed to be because of chemotherapy as she had no signs or symptoms of infection.

On day 5 of chemotherapy, the patient complained of a throbbing frontal headache only partially relieved by nonsteroidal anti-inflammatory drugs (NSAIDs) and morphine. A CT scan of the head was ordered which was normal. Neurology was consulted for persistent headache, and they deemed the headache was due to chemotherapy as her findings were not consistent with idiopathic intracranial hypertension. On day 9 of chemotherapy, her ATRA dose was decreased to 56 mg daily instead of twice daily due to increasing WBC count and concern for DS. Despite decreasing the dose of ATRA, her WBC count continued to increase. On day 10 of chemotherapy, dexamethasone 10 mg twice a day was started for prophylaxis of DS due to an uprising WBC count. On day 12, the WBC count reached 37.6 K/µL. She started complaining of right upper quadrant abdominal pain and stated she had not had a bowel movement in a week. Her liver function tests showed aspartate transaminase (AST) of 102 units/L and alanine transaminase (ALT) of 100 units/L, improving the next day with AST 54 units/L and ALT 73 units/L. An abdominal ultrasound was ordered which showed hepatomegaly with fatty infiltration. Her abdominal pain was thought to be due to constipation and was relieved by laxatives. ATRA and ATO were stopped on day 13 due to increasing WBC count and concerns for worsening DS. Blood cultures drawn on day 13 started growing gram-negative rods. 

On day 14 of chemotherapy, a rapid response was called as the patient was complaining of respiratory distress with severe abdominal pain. The patient was found to be wheezing on auscultation requiring 5 liters of oxygen to maintain oxygen saturation, with a blood pressure of 128/72 mmHg, heart rate of 160 bpm, and body temperature of 38.8°C. ECG showed sinus tachycardia. Chest X-ray showed pulmonary vascular congestion. A dose of diphenhydramine 25 mg was given for possible anaphylaxis, and the patient was transferred to the intensive care unit (ICU) and placed on a high-flow nasal cannula. Labs done after ICU transfer showed a WBC count of 93.2 K/µL, Hb 9 g/dL, platelets 67x10^3^/uL, INR 2, creatinine 1.55 mg/dL, ALT 58 units/L, AST 44 units/L, bicarbonate 10 mEq/L, anion gap 22 mmol/L, troponin 0.012 μg/mL, LDH 1351 units/L, and lactic acid 13.5 mmol/L. A TTE showed LVEF 25-30% with hypokinesis of septal myocardium, compared to LVEF 60-65% on admission. A small pericardial effusion was also identified but without any evidence of tamponade. The inferior vena cava was found to be dilated, and a right atrial pressure of 15 mmHg was noted, both consistent with volume overload. The patient was given 40 mg of furosemide and started on vancomycin and piperacillin-tazobactam for broad antibiotic coverage, which was later escalated to meropenem, amikacin, and micafungin to expand antimicrobial coverage as blood cultures drawn on day 13 were growing gram-negative rods. Dexamethasone was continued at 10 mg every 12 hours, and an additional dose of methylprednisolone 250 mg IV was given for DS. Two additional doses of methylprednisolone 125 mg were given shortly after.

The patient ended up going into a pulseless electrical activity (PEA), a code blue was called, and a return of spontaneous circulation (ROSC) was obtained after one round of cardiopulmonary resuscitation (CPR). Endotracheal intubation was done, and the patient was started on propofol for sedation. Twenty minutes after the first PEA, another code blue was called, and the patient was found to be in PEA again and ROSC was obtained after two rounds of CPR. She required multiple vasopressors including norepinephrine, vasopressin, phenylephrine, and epinephrine but continued to have poor perfusion with severe skin mottling, oliguria, and persistent lactic acidosis. A family discussion was held due to the poor prognosis from multifactorial shock, and the patient's code status was changed to do not resuscitate (DNR). Soon afterward, the patient suffered another cardiac arrest and passed away. 

## Discussion

The mechanism of DS is incompletely understood but involves a large number of differentiating promyelocytes induced by ATRA and ATO to have increased adhesion molecules that promote infiltration of various organs. A systemic inflammatory response leads to increased cytokine expression and chemokine production that causes endothelial damage leading to capillary leak syndrome and microcirculation occlusion [4]. There are no diagnostic criteria for DS which are further complicated by a lack of specific biomarkers to confirm the diagnosis of DS. Leukocytosis, anemia, thrombocytopenia, and coagulopathy are common in DS, but these abnormalities can also be due to underlying APL. DS can mimic infection, congestive heart failure, anaphylaxis, and thromboembolism, and these conditions have to be effectively ruled out. The diagnosis is made clinically with associated signs and symptoms such as dyspnea with interstitial pulmonary infiltrates, acute kidney injury (AKI), pleuropericardial effusion, hypotension, peripheral edema, hyperbilirubinemia, and musculoskeletal pain [5]. DS has a bimodal distribution peaking at week 1 and then at week 3 [5]. However, our patient's presentation worsened rapidly on day 14, highlighting the unpredictable nature of DS. Physicians managing patients with APL undergoing treatment with ATRA and ATO should remain vigilant for such signs and symptoms throughout chemotherapy.

The APL0406/2013 study suggested administering DS prophylaxis using prednisone at 0.5 mg/kg/day from day 1 throughout the induction period [6]. Lo-Coco protocol during a phase 3, multicenter trial comparing ATRA plus chemotherapy with ATRA plus ATO in patients with APL used DS prophylaxis with prednisone at a dose of 0.5 mg per kilogram of body weight per day from day 1 until the end of induction therapy, highlighting the importance of prophylaxis with steroids [7]. In cases of marked hyperleukocytosis when ATO-based regimens are used, cytoreductive agents, such as hydroxyurea, anthracyclines, idarubicin, and gemtuzumab ozogamicin, are often used. However, Programa Español de tratamientos en Hematología (PETHEMA) reported that steroid prophylaxis showed a modest reduction of DS incidence, but no differences in DS-related mortality for APL treated with ATRA [8]. In the LPA99 trial, the systematic use of prednisone prophylaxis showed no effect on reducing mortality from DS compared with a selective use of dexamethasone prophylaxis in patients with WBC count greater than 5×109/L in the LPA96 trial [8].

As DS can present suddenly and can be very severe leading to death as in this case, we believe the benefits of prophylactic corticosteroids outweigh the risks. There are no randomized trials of early treatment with systemic corticosteroids versus delayed therapy for DS in APL. This case report calls for such randomized trials to establish effective and precise guidelines to reduce mortality from DS and include the administration of corticosteroids as prophylaxis for DS in APL treatment with ATRA and ATO. Corticosteroids do not reduce the mortality from DS, but perhaps earlier administration of prophylactic corticosteroids and cessation of ATRA and ATO can lead to better outcomes with reduced severity of DS. 

## Conclusions

DS is a potentially fatal complication of APL induction chemotherapy with ATRA and ATO that follows an unpredictable and complex course. In our case, corticosteroids were started on day 10, and ATRA and ATO were discontinued on day 13. This case highlights the importance of timely recognition of DS and early prophylactic corticosteroid administration with prompt discontinuation of ATRA and ATO to improve outcomes. It further emphasizes the need for more research and randomized trials to establish treatment protocols and a uniform corticosteroid administration schedule incorporated into guidelines to improve morbidity and mortality from DS. 
